# Fabrication of Green PVDF/TiO_2_ Composite Membrane for Water Treatment

**DOI:** 10.3390/membranes15070218

**Published:** 2025-07-21

**Authors:** Shuhang Lu, Dong Zou

**Affiliations:** School of Environmental Science and Engineering, Nanjing Tech University, Nanjing 211816, China; lushuhang@njtech.edu.cn

**Keywords:** green solvent, PVDF/TiO_2_ membranes, phase inversion method, water treatment

## Abstract

PVDF/TiO_2_ composite membranes show some potential to be used for water treatment as they combine the advantages of polymers and ceramics. However, conventional PVDF-based composite membranes are always fabricated by using conventional toxic solvents. Herein, PolarClean was used as a green solvent to fabricate PVDF/TiO_2_ composite membranes via the phase inversion method. In this process, Pluronic F127 was used as a dispersion agent to distribute TiO_2_ particles in the PVDF matrix and to serve as a pore former on the membrane surface. TiO_2_ particles were well distributed on the membrane surface and bulk. TiO_2_ particles in the PVDF matrix enhanced the mechanical strength and hydrophilic characteristics of the resulting composite membrane, facilitating water transport through the composite membranes and enhancing their water permeability. Membrane microstructures and mechanical strength of the composite membranes were finely tuned by varying the PVDF concentration, TiO_2_ concentration, and coagulation bath temperature. It was demonstrated that the resulting green PVDF/TiO_2_ composite membrane showed a high water permeance compared with those using conventional toxic solvents in terms of its small pore size. In addition, the particle rejection of green PVDF/TiO_2_ membrane showed a 99.9% rejection rate in all the filtration process, while those using NMP showed 91.1% after 30 min of filtration. The water flux was similar at 121 and 130 Lm^−2^h^−1^ for green and conventional solvents, respectively. This work provides important information for the future application of sustainable membranes.

## 1. Introduction

Poly(vinylidene fluoride) (PVDF) is one of the most commonly used polymer membrane materials for water treatment due to its thermal resistance and chemical stability [1,2,3,4]. However, the hydrophobic nature of PVDF membranes has a detrimental effect on water transport in water treatment systems. On the other hand, pure PVDF membranes are fouled easily by organic contaminants present in the wastewater, greatly decreasing the water permeance through the membrane. To solve this problem, many studies have begun to adopt surface modification [5], nanoparticles (NPs)-embedded composite membranes [6,7], and other effective methods to mitigate the fouling of PVDF membranes. Among the inorganic NP-embedded approaches (Al_2_O_3_, SiO_2_, TiO_2_, ZrO_2_, and others), TiO_2_ particles have been widely used as raw materials to fabricate composite membranes in recent years, as TiO_2_ NPs possess higher hydrophilic properties and photocatalytic activity than other inorganic particles [8]. The composite membrane can form a hydration layer on the membrane surface, effectively alleviating adsorption of pollutants on the membrane surface to decrease membrane fouling [9]. However, the mixing process of solvents and inorganic particles is a problem [6,10]. Inorganic particles readily agglomerate in polymer solutions, negatively impacting the performance of the final composite polymer membranes. Ultrasonic treatment or coating modification has been used commonly to disperse these particles in the dope solution. Galiano et al. [11] first dispersed TiO_2_ particles in water via a stirring process, followed by an ultrasonic treatment for 30 min to achieve a relatively stable TiO_2_ dispersion. Then, the particles were mixed with polymers and solvents to form a dope solution.

Currently, non-solvent-induced phase separation (NIPS) and thermally-induced phase separation (TIPS) are the two major methods for fabricating polymer membranes. Regardless of the process, polymers need to be dissolved in solvents first. Typically, *N*-methyl pyrrolidone (NMP) or *N,N*-dimethylacetamide (DMAc) has been used to dissolve PVDF powders and to fabricate membranes [12,13]. However, NMP or DMAc is toxic to humans and bad for the environment [14,15]. For example, NMP has been banned by the European Union due to toxicity since 2020 [16]. Without these widely used conventional solvents, it is unclear how PVDF membranes can be fabricated. Many membrane scientists realize that the present membrane fabrication processes are not “green” due to the use of toxic solvents [17,18]. Therefore, they have started to employ “harmless solvents” or begun to fabricate polymer membranes without solvents [19]. Among the reported nontoxic solvents, PolarClean is popular because it is biodegradable and has no or a very slight effect on human health and the environment [20]. Furthermore, it is nonflammable and has very low vapor pressure [21]. Compared with conventional solvents, such nontoxic solvents are particularly favorable for membrane fabrication. In our previous work, PolarClean, as a green solvent, was used for the fabrication of macroporous PVDF membranes via the phase inversion method for membrane distillation [22,23], demonstrating that this green solvent can be used for polymer membrane materials.

**Figure 1:**
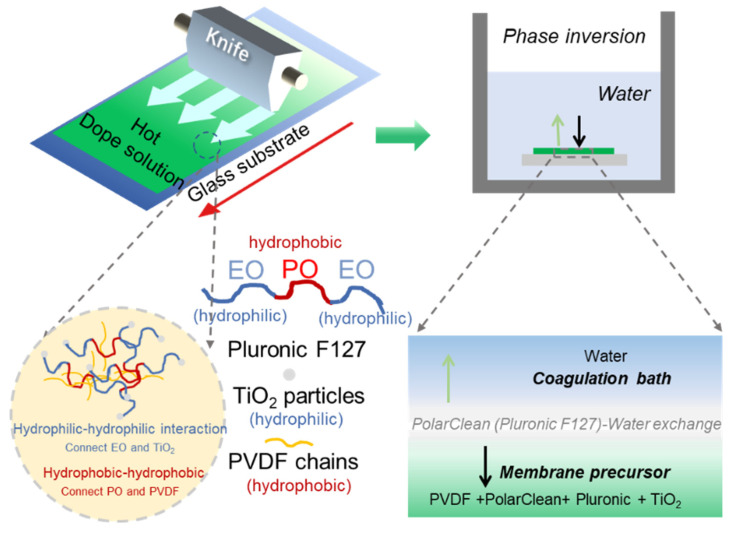
The fabrication process of PVDF/TiO_2_ membrane by using PolarClean as a green solvent.

In this work, we use PolarClean as a green solvent for the fabrication of high-performance PVDF/TiO_2_ composite membranes (see Figure 1 for the detailed fabrication process). Pluronic F127 (PF127) was employed to well distribute the TiO_2_ particles in the dope solution and to induce pore structures on the membrane surface. The coagulation temperature, PVDF concentration, and TiO_2_ content on the membrane performance were investigated to seek the possibility for the fabrication of high-performance PVDF/TiO_2_ by using green solvent. Finally, the separation performance of the resulting green membrane was also compared with that of the NMP solvents.

## 2. Experimental

### 2.1. Materials

PVDF powders (Solef 1015) and PolarClean were provided by Solvay Specialty polymers (Bollate, Italy). Pluronic F127, TiO_2_ particles with an average particle size of 20 nm, were purchased from Sigma-Aldrich Chemical Co. (Milwaukee, WI, USA). Deionized water (DI) was produced by a Milli-Q system (Millipore^®^, Burlington, MA, USA).

### 2.2. Membrane Fabrication

#### 2.2.1. PolarClean/PVDF Membranes

Dope solutions with PVDF concentrations of 10–20 wt% and a Pluronic F127 (PF127) content of 5 wt% were fabricated at 140 °C with stirring for 2.5–3 h. A homogenous solution was obtained and degassed for 1 h at 140 °C, and the solution was then cast onto a glass plate [23]. After that, the membranes were immediately immersed in a water coagulation bath immediately for ~36 h at 20 or 60 °C (refreshing the water every 12 h). Then, the membrane was washed with ethanol for 36 h to remove the remaining PolarClean solvent. Finally, these membranes were immersed in hexane to extract the ethanol and were dried to preserve the pore structures for characterization.

#### 2.2.2. PolarClean/PVDF/TiO_2_ Membranes

The fabrication process of the composite membranes is similar to that of PVDF membranes mentioned in Section 2.2.1. After PF127 was dissolved in PolarClean at 50 °C for 2 h, 0.5–2 wt% TiO_2_ particles were added, and the mixture was stirred for 12 h at 50 °C.

#### 2.2.3. NMP/PVDF and NMP/PVDF/TiO_2_ Composite Membranes

PVDF powders were dissolved in NMP at 50 °C for 24 h to form a homogenous solution (15 wt%). The solution was held at 50 °C for 6 h to remove any air bubbles in the dope solution. Then, as mentioned in Section 2.2.1, the dope solution was cast onto a glass plate and immersed in water for 24 h. During this period, the water was replaced three times. Membranes were dried in an oven at 50 °C for 12 h. The fabrication process of the NMP/PVDF/TiO_2_ dope solution was similar to that used for the NMP/PVDF solution. However, TiO_2_, PF127, and NMP were mixed at 50 °C for 12 h in advance for composite membranes. Then, PVDF powders were added and stirred for 24 h. The other fabrication processes were similar to those of NMP/PVDF membranes.

### 2.3. Characterization

A scanning electron microscope (FESEM S4800, Hitachi, Tokyo, Japan) was used to characterize the surface and cross-sectional structure of membranes. Before measurement, the samples were sprayed with platinum at 15 mA for 120 s using a platinum sputter (Hitachi E-1045, Tokyo, Japan). A universal testing machine (AGS-J 500N, Shimadzu, Kyoto, Japan) was used to measure the mechanical strength, including the tensile strength and elongation at the break. Membrane samples were cut into a dumbbell-like shape with an effective area of 2 mm × 10 mm using a standard mold with an elongation rate set to 10 mm min^−1^. A capillary flow porometer (CFP-100-AE, Porous Materials Inc., Ithaca, NY, USA) was used to measure the pore size distribution of the PVDF/TiO_2_ composite membranes. These flat sheet membranes were immersed in galwick (surface tension of 15.9 dynes/cm) before measurement.

The overall porosity of the PVDF-based membranes was calculated using Equation (1), where *W*, *L*, and *H* represent the width, length, and height of the membranes, respectively. *M* represents the mass of the sample, and *ρ* represents the density of the PVDF materials.

(1) ε=1−MρW×L×H

A cross-flow filtration device was used to measure the water permeance of these membranes. The fabricated membranes were immersed in EtOH solution for at least 30 min to open the permeation pathway. Then, the membranes were placed in a metal model with an effective area of 9.6 cm^2^. After that, the membranes were used to filter DI water for 30 min, and then the water permeance was calculated using Equation (2). Here, *J* is water permeance (Lm^−2^h^−1^bar^−1^), *V* is water volume (L), *A* is effective membrane area (m^2^), *t* is filtration time (h), and *P* is trans-membrane pressure (bar). A solid/liquid system containing TiO_2_ nanoparticles (600 ppm) with an average particle size of 20 nm was employed to characterize the separation performance compared with conventional systems.

(2) $$J=\frac{V}{A\cdot t\cdot P}$$

## 3. Results and Discussion

### 3.1. Effects of TiO_2_ Content and Coagulation Temperature

In this work, TiO_2_ was used to enhance the hydrophilic performance of the PVDF matrix while Pluronic acted as a pore formation agent during the phase inversion process. TiO_2_ and Pluronic can cooperate with each other to finely tune the microstructure of the PVDF membrane. Upon mixing TiO_2_ and PF127 in the PVDF dope solution, water was used as a coagulation medium in this phase inversion process. The TiO_2_ content in the dope solution and coagulation temperature were investigated and discussed here, as the diffusion rate of the solvents and water during the coagulation of the membrane has a great impact on the microstructure of the resulting membrane.

**Figure 2:**
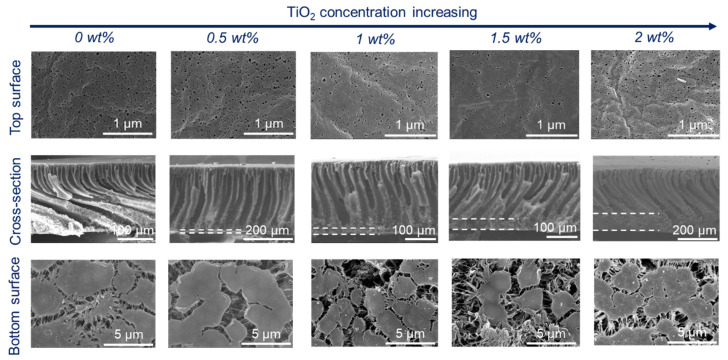
Membrane microstructures of PVDF/TiO_2_ composite membranes with different TiO_2_ contents: 15 wt% PVDF content, 5 wt% PF127, and coagulation temperature of 20 °C.

**Figure 3:**
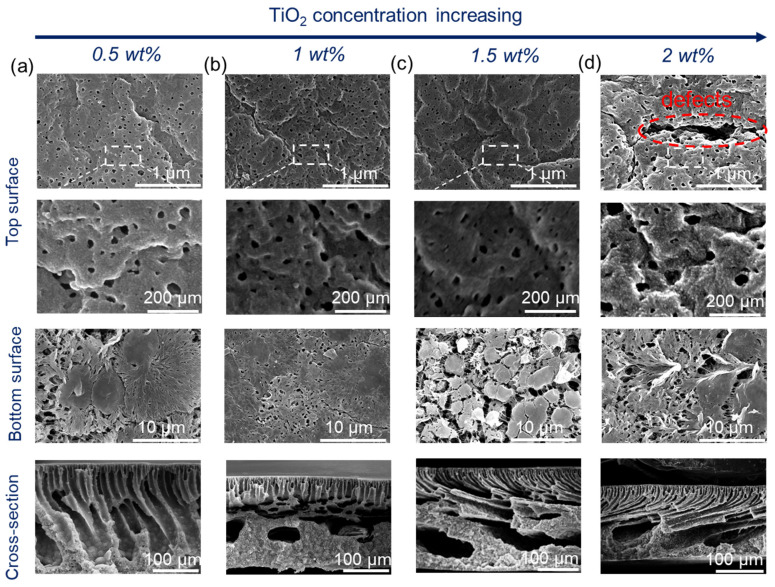
Membrane microstructures of PVDF composite membranes with different TiO_2_ contents: 15 wt% PVDF, 5 wt% PF27, and a coagulation temperature of 60 °C. (**a**) 0.5 wt%, (**b**) 1 wt%, (**c**) 1.5 wt%, and (**d**) 2 wt%.

The content of PF127 was controlled at 5 wt%, while TiO_2_ content was controlled from 0 wt% to 2 wt% when the coagulation temperature was set to 20 °C (Figure 2) or 60 °C (Figure 3). The cross-sectional structure of the composite membrane is mainly composed of a finger-like structure when the coagulation bath temperature is 20 °C. That was because the PF127 and a certain amount of TiO_2_ enhanced the hydrophilicity of the PVDF matrix, which changed delayed demixing to instantaneous demixing compared with pure PVDF membranes [24].

With an increase in TiO_2_ content, the viscosity of the dope solution increased [25] and thus delayed the phase separation. Therefore, the thickness of the finger-like structure decreased slightly (Figure 2). Upon increasing the coagulation temperature to 60 °C (Figure 3), macrovoids in the membrane cross-section were observed because high temperature accelerated the phase inversion rate and PF127 removal rate in the water. The membrane surfaces prepared at both 20 °C and 60 °C displayed porous structures. However, the membrane surface showed obvious defects when the coagulation temperature was set to 60 °C and the TiO_2_ content was 2 wt% (Figure 3). This was because a high TiO_2_ concentration (2 wt%) can result in the aggregation of NPs on both the bulk and surface of the composite membrane [26,27]. In addition, high temperature can promote the aggregation of PF127 micelles as it is temperature-independent. The number of PF127 micelles increases with the temperature increase, which is believed to result in a larger pore size in this system [28]. These TiO_2_ NPs will be brought into the water bath by these micelles, and the position of these aggregated TiO_2_ NPs will be absent, thus resulting in defects on the membrane surface. All the bottom surfaces were highly porous, regardless of the coagulation temperature (20 °C or 60 °C).

**Figure 4:**
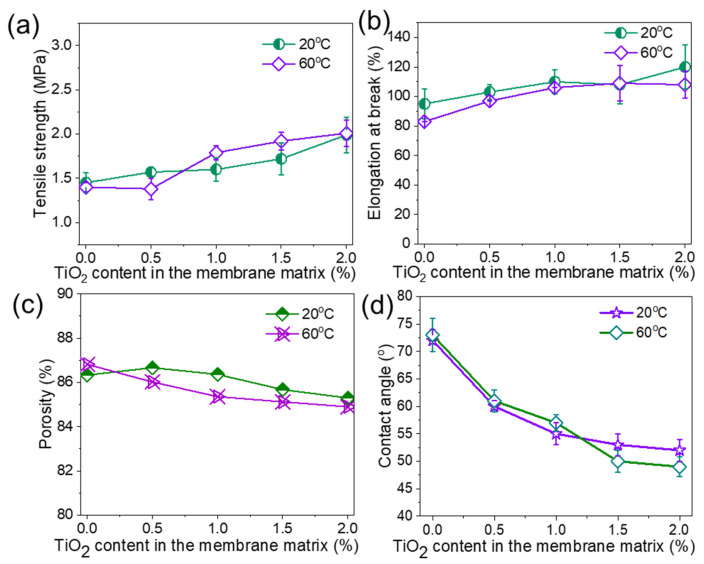
Properties of PolarClean/PVDF/TiO_2_ composite membranes at different TiO_2_ contents and coagulation temperatures. (**a**) Tensile strength, (**b**) elongation at break, (**c**) porosity, and (**d**) water contact angle. PVDF concentration is 15 wt%.

The physical properties and water transport performances of PVDF/TiO_2_ composite membranes are shown in Figure 4. Tensile strength and elongation at break slightly increased with TiO_2_ content. The main reason for this phenomenon is that the applied external force can be distributed on the polymer chains and NPs. Thus, the tensile strength can be improved, as the membrane samples do not break easily under higher forces [29,30]. In addition, the interaction between the PVDF and NPs enhances the rigidity and thus improves the mechanical strength, and these NPs could act as cross-linking agents to connect the polymer to the final membranes [31]. The porosity of these membranes slightly decreased from ~87% to 85% with an increase in TiO_2_ content (Figure 4c) due to the presence of these particles in part of the pore voids. Figure 4d shows the contact angles of the neat PVDF and composite membranes. Similar to mechanical strength and porosity, coagulation temperature had no obvious effect on surface hydrophilicity, as the hydrophilic performance of the composite membranes was mainly controlled by TiO_2_ concentration. The contact angle of the neat PVDF membranes was ~72°. As the content of TiO_2_ NPs increased, the contact angle decreased to ~52°.

**Figure 5:**
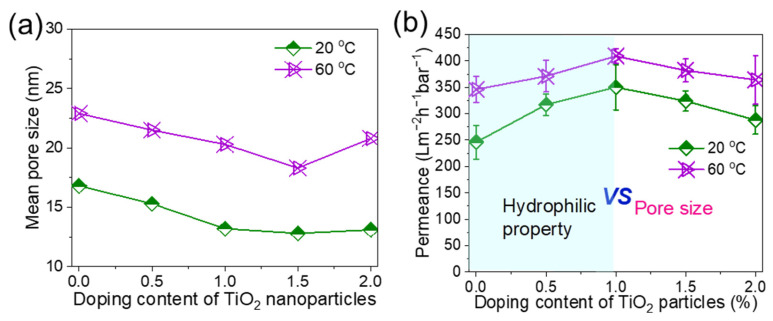
Performance of PolarClean/PVDF/TiO_2_ composite membranes at different TiO_2_ contents and coagulation temperatures. (**a**) Mean pore size, and (**b**) water permeance. PVDF concentration is 15 wt%.

The effects of coagulation temperature and TiO_2_ content on the pore size and water permeance are presented in Figure 5a,b. In general, lower coagulation temperature and higher TiO_2_ content decreased the pore size [26,27] of the membrane. However, when the TiO_2_ content was increased to about 2 wt%, TiO_2_ aggregated, leading to an increase in membrane pore size. It was particularly obvious when the coagulation temperature was at 60 °C, which was consistent with the SEM images in Figure 3. The water permeance at both 20 °C and 60 °C increased and then decreased as the TiO_2_ content exceeded 1 wt%. This is because the hydrophilic aspect and pore size effect compete with each other [32], where the hydrophilic nature of TiO_2_ first enhances the water permeance while the decreased pore size dominates over 1 wt% TiO_2_ content. Therefore, as shown in Figure 5b, the water permeance increased to ~350 (20 °C) and ~ 409 Lm^−2^h^−1^bar^−1^ (60 °C) and then decreased to 302 and 384 Lm^−2^h^−1^bar^−1^, respectively, due to the synergistic effects of pore size and hydrophilic performance.

### 3.2. Effect of PVDF Concentration

**Figure 6:**
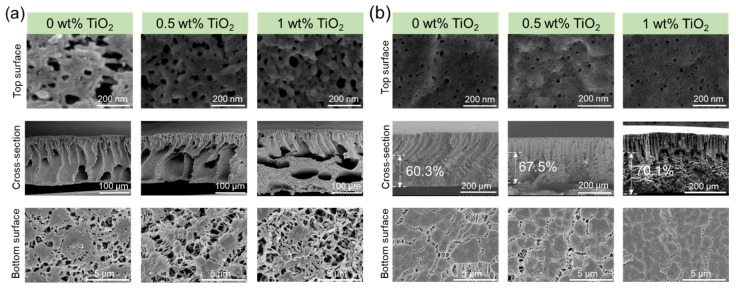
Microstructure of PolarClean/PVDF/TiO_2_ membranes. (**a**) PVDF concentration of 10 wt% (mass ratio of PF127 to PVDF is 50%), and (**b**) PVDF concentration of 20 wt% (mass ratio of PF127 to PVDF is 25%).

Usually, the polymer content in a dope solution greatly impacts the final performance of the polymer membrane. That is because the polymer content increases the solution viscosity, which affects the phase inversion processes. Accordingly, the porosity, water permeability, mechanical strength, and other parameters were investigated in detail (Figure 6 and Table 1). It was clear that the membrane surface, cross-sectional structure, and bottom surface were different. When the PVDF concentration in the dope solution was 10 wt% (Figure 6a), the finger-like structures in the cross-section were larger than those prepared from 15 wt% PVDF content, as in Figure 2. We observed a transition from a finger-like structure to a combination of finger-like and sponge-like structures as TiO_2_ content increased to 1 wt%.

The structures of the composite membranes with the 20 wt% PVDF solution were different from those with 10 wt% PVDF content. The cross-section of the 20 wt% PVDF membrane (Figure 6b) showed a combination of spherulitic morphology and finger-like morphology, and the proportion of spherulitic thickness increased from 60.3% to 70.1% when TiO_2_ content increased to 1 wt%. Further increasing the PVDF concentration to 25 wt%, the spherulitic structure is also very obvious. Accordingly, the mechanical strength of the membranes prepared at 20 wt% PVDF content (Table 1) was higher than that from 10 wt% polymer concentration. This was attributed to a loss of macrovoids, which enhanced the membrane connectivity among the polymer matrix [33]. Furthermore, the cross-sectional structure explained why the porosity increased at lower polymer concentration (with porosity above 90% at 10 wt% polymer content).

Membranes made with low PVDF content showed larger mean pore sizes. For example, the pore size of membranes with 10 wt% PVDF concentration ranged from 30.3 to 25.6 nm, while those with 20 wt% PVDF showed a pore size of 6–10 nm in Table 1. PF127 content was the same in the dope solutions with 10 or 20 wt% PVDF (5 wt%). Therefore, the mass ratio of PF127 to PVDF at 10 wt% PVDF content was 0.5, while that of PF127 to PVDF at 20 wt% was 0.25. The larger pore size of the membrane can be attributed to the aggregation behaviors of Pluronic at higher concentration [34], which possibly turned the F127 particles into spheres in larger sizes and diminished the surface contact of F127 with PVDF [34]. Thus, it is easier to be washed out by water and a larger pore size is formed [35]. This means that the mean pore size of the membrane from 10 wt% PVDF was much larger. The pure water permeance ranged from ~1000 to 1200 Lm^−2^h^−1^bar^−1^, from ~95 to ~110 Lm^−2^h^−1^bar^−1^ and from 35 to 48 Lm^−2^h^−1^bar^−1^ for membranes prepared from 10, 20, and 25 wt% PVDF, respectively.

### 3.3. Distribution of TiO_2_ Particles

**Figure 7:**
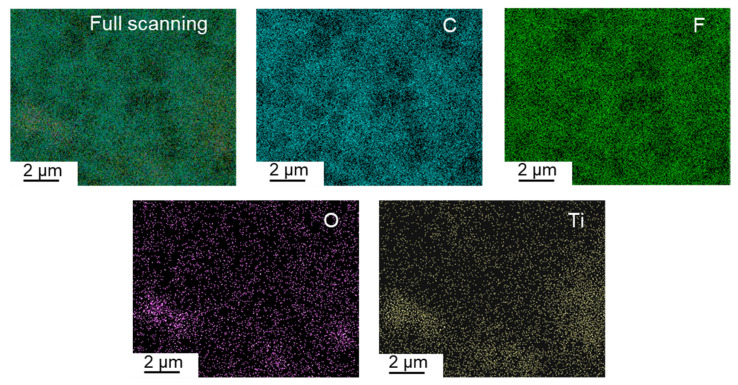
The elemental distribution of TiO_2_ particles on the membrane surface.

Well-dispersing TiO_2_ particles in the polymer matrix is very important for composite membranes because serious aggregation of TiO_2_ could destroy the integrity of the membrane and affect membrane porosity and resulting performance. Figure 7 shows the EDX-mapping of the PVDF/TiO_2_ composite membrane surface, demonstrating that the TiO_2_ particles are well dispersed in the PVDF matrix. There are two main reasons for this phenomenon. First, PolarClean is miscible with water, and TiO_2_ is very hydrophilic. Therefore, PolarClean and TiO_2_ could be dispersed well, although they are not completely miscible. Second, PF127 is a linear molecule with hydroxyl groups at both ends that form non-covalent interactions with TiO_2_ [36], resulting in good dispersion of PF127 and TiO_2_ in PolarClean. After dissolving PVDF powders in the PolarClean/TiO_2_/PF127 system, PVDF chains can connect with the methyl groups via hydrophobic interactions (van der Waals interaction). As a consequence, a well-dispersed solution can be formed at 140 °C.

**Figure 8:**
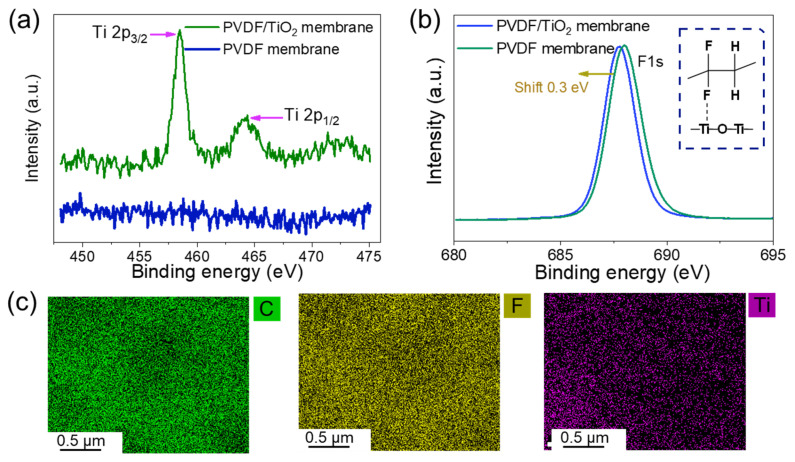
The stable performance of the PVDF/TiO_2_ composite membranes. The XPS results of (**a**) Ti and (**b**) F of the composite membranes and PVDF membranes, and (**c**) the elemental distribution on the membrane surface after pure water filtration. DI water was used in this test with a transmembrane pressure of 0.5 bar and a filtration time of 24 h.

Here, we adopted XPS characterizations to measure the bonding strength of TiO_2_ particles and PVDF. Figure 8a shows the Ti XPS results of the PVDF membrane and PVDF/TiO_2_ composite membrane. As shown, only Ti peaks are observed in the composite membrane and are divided into Ti 2p_1/2_ and Ti 2p_3/2_ peaks situated at 464.38 eV and 458.48 eV, respectively. These peaks are assigned to Ti (IV), implying the existence of TiO_2_ nanoparticles in the composite membrane [37]. Figure 8b shows the XPS curves of the fluorine (F) atom from the two membranes. Note that the F peak of the composite membrane shifted about 0.3 eV toward the high binding energy region compared with the neat PVDF membranes. The shift demonstrated a coordination between F and Ti atoms [38]. Ye et al. [39] showed similar XPS results. As F is a strongly electro-negative atom, coordination bonds between F and Ti can act as electron-trapping sites. The elemental distribution on the membrane surface after filtering pure water for 24 h is shown in Figure 8c. The TiO_2_ particles on the membrane surface indicate stable bonding strength between the PVDF chains and Ti NPs even after 24 h of water permeation.

### 3.4. Performance Comparison of PVDF/TiO_2_ Membrane from NMP

**Figure 9:**
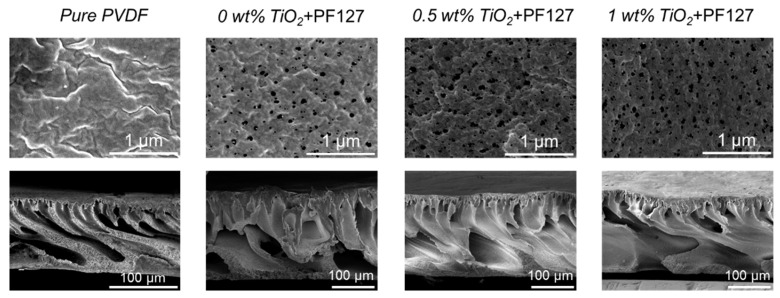
Membrane morphologies of NMP/PVDF membranes. PF127 concentration is 5 wt%.

Conventional PVDF/TiO_2_ composite membranes usually use NMP or DMAc as solvents [27,40,41,42]. Here, we also adopted NMP to fabricate NMP/PVDF/TiO_2_ membranes for comparison using the same fabrication conditions employed for PolarClean. Figure 9 shows the microstructure of the PVDF membranes prepared from the NMP system. The surface of the pure NMP/PVDF membrane was slightly dense. After adding PF127 and TiO_2_ NPs, the membrane surface showed a highly porous structure. Cross-sectional images of the NMP/PVDF/TiO_2_ membranes showed finger-like structures.

**Figure 10:**
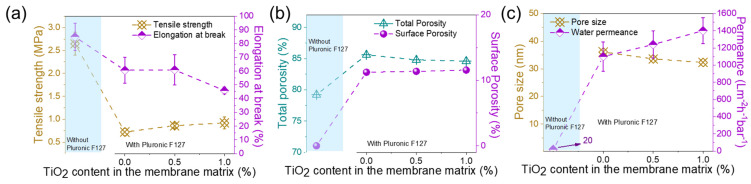
(**a**) tensile strength and elongation at break, (**b**) porosity, and (**c**) pore size and water permeance. PF127 concentration is 5 wt%. Surface porosity is calculated by the Image J2 software.

Figure 10 demonstrates that these membranes have a poorer tensile strength (ranging from 0.72 to 0.92 MPa) and elongation at break (60% to 67%) than those fabricated from PolarClean. The tensile strength for the PolarClean/PVDF/TiO_2_ system increased from 1.45 MPa to 1.99 MPa and showed elongation from 95% to 120%, as in Figure 4a,b. The porosity of PVDF membranes from NMP ranged from 85.6% to 84.6% (Figure 10b), which was similar to that of those from PolarClean (ranging from 86.7% to 85.3% in Figure 4c).

However, the pore size and permeance were very different (Figure 10c). The pore sizes of the NMP/PVDF/TiO_2_ composite membranes (36.1 nm to 32.3 nm) were significantly larger than those of PolarClean/PVDF/TiO_2_ membranes (16.8 nm to 10.6 nm). Therefore, the permeance of the composite membrane prepared from NMP was large, i.e., above 1200 Lm^−2^h^−1^bar^−1^. The water permeance of the composite membrane prepared from the PolarClean system was between 280 and 350 Lm^−2^h^−1^bar^−1^. According to the Hagen–Poiseuille equation (Equation (3)), if the pore size is twice as large, the water permeance of the membrane is four times larger. Comparing PolarClean or NMP-containing PVDF/0.5 wt% TiO_2_, the mean pore size and water permeance of NMP/PVDF/TiO_2_ membranes were 33.6 nm and 1240 Lm^−2^h^−1^bar^−1^, respectively. If the pore size of such a membrane decreased to 15.3 nm, the permeance was estimated to be ~260 Lm^−2^h^−1^bar^−1^ based on Equation (3) (ε/32 μLτ is considered a constant in this case). The result is lower than that of the PolarClean/PVDF/0.5 wt%TiO_2_ membrane (water permeance of 317 Lm^−2^h^−1^bar^−1^ and pore size 15.3 nm), demonstrating that the permeance of these composite membranes from PolarClean is comparable to that from NMP at the same experimental conditions, and they possess high mechanical strength.

(3) $$J=\frac{F}{\Delta P}=\frac{\varepsilon }{32\mu L\tau }\cdot {d_{m}}^{2}$$

Here, *d_m_* represents membrane pore size, ε represents membrane porosity, τ represents tortuosity, *μ* represents liquid viscosity, *L* represents membrane thickness, *F* represents water flux, Δp represents trans-membrane pressure, and *J* represents water permeance.

Table 2 lists PVDF/TiO_2_ membranes prepared from conventional solvents (i.e., DMAc, NMP, and DMF) and the present PolarClean solvents in this work (with 15 wt% PVDF). The membranes presented in this work exhibited a higher permeance compared with those of other published works. This demonstrates that PolarClean, as a green solvent, has great potential for the fabrication of PVDF/TiO_2_ composite membranes.

### 3.5. Separation of Particle-Laden Waste Water

**Figure 11:**
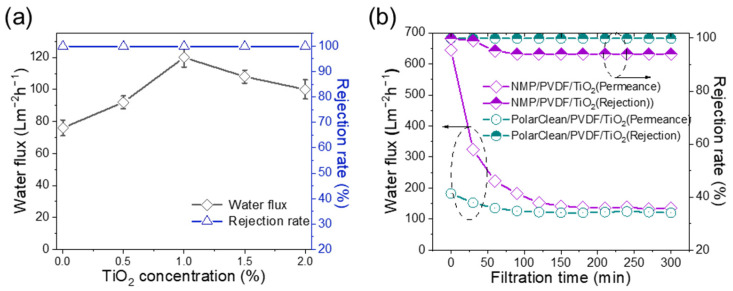
The separation performance of PVDF/TiO_2_ membranes by varying PVDF and TiO_2_ concentration. (**a**,**b**) Rejection and comparison of PolarClean/PVDF/TiO_2_ membrane and NMP/PVDF/TiO_2_ membrane in the separation of solid–liquid system (trans-membrane pressure is 0.5 bar).

Solid–liquid suspension containing NPs was used to characterize the separation performance of PVDF (15%)/TiO_2_ membranes (Figure 11a). It can be seen that the rejection rate of the membranes was stable above 99%, and the stable permeance is maximum when the TiO_2_ concentration is 1 wt%. Furthermore, we employed PVDF/TiO_2_ (1 wt%) membranes fabricated from PolarClean and NMP for comparison. As is shown in Figure 11b, the NPs in the model wastewater are prone to block the pore channel of the NMP/PVDF/TiO_2_ membrane, which will greatly increase the filtration resistance. However, the pore size of PolarClean/PVDF/TiO_2_ membranes (~12 nm) is smaller than the particle size of NPs (~20 nm) in the wastewater. Therefore, these NPs are mainly deposited on the membrane surface, which will not increase the filtration resistance greatly. In addition, the rejection rate of the PolarClean/PVDF/TiO_2_ membrane (99.9%) is higher than that of NMP/PVDF/TiO_2_ membranes (91.1%) after a 30 min filtration process. Notice that the rejection rate of NMP/PVDF/TiO_2_ is higher at the beginning. That is because these NPs in the wastewater are easily absorbed into the pore channels, and then go through the membrane to the feed solution as the filtration time increases. In general, such green PolarClean/PVDF/TiO_2_ membranes have a great potential to separate wastewater containing nanoparticles.

## 4. Conclusions

PolarClean was used to fabricate PVDF/TiO2 composite membranes for water treatment. Since PolarClean has a limited solubility compared with conventional solvents, PVDF was dissolved at 140 °C for 3 h to form a uniform dope solution and fabricate PVDF/TiO_2_ membranes via phase inversion. The microstructure of the membranes, pore size, mechanical strength, and water permeance of the composite membrane were affected by the PVDF concentration in PolarClean, coagulation temperature, and TiO_2_ doping content. In addition, the binding energy and dispersion process of the TiO_2_ in the PVDF solution with the aid of PF127 was investigated in detail. Compared with the NMP (DMAc)/PVDF/TiO_2_ system reported in the literature, the present PolarClean/PVDF/TiO_2_ composite membrane showed competitive performance. By varying the PVDF concentration, TiO2 concentration, and coagulation temperature, the green PVDF/TiO_2_ membranes can be employed in water treatment. In the separation of particle-laden wastewater, the stable permeance of NMP/PVDF membrane (130 Lm^−2^h^−1^) is similar to that of PolarClean/PVDF membrane (121 Lm^−2^h^−1^), demonstrating that PolarClean (an alternative green solvent) is suitable to replace conventional solvents like NMP, DMAc, and others for composite membranes.

## Figures and Tables

**Table 1:** The performance of PVDF/TiO_2_ composite membranes at different polymer contents (with PF127 content of 5 wt%).

Polymer Content (%)	TiO_2_ Doping Content (%)	Tensile Strength (MPa)	Elongation at Break (%)	Porosity (%)	Mean Pore Size (nm)	PWP (Lm^−2^h^−1^bar^−1^)
10	0	0.93 ± 0.16	77.49 ± 10.2	90.92	30.3	1025 ± 120
10	0.5	1.12 ± 0.10	108.7 ± 25.2	91.35	27.2	1100 ± 98
10	1	1.32 ± 0.13	118.3 ± 18.0	91.20	25.6	1150 ± 170
20	0	1.99 ± 0.08	115 ± 20.5	79.14	10.2	95 ± 10
20	0.5	2.15 ± 0.12	126 ± 5.9	79.10	6.3	105 ± 22
20	1	2.28 ± 0.20	130 ± 17.6	79.03	6.0	108 ± 20

**Table 2:** Performance of PVDF/TiO_2_ composite membranes compared with those of previous studies using conventional solvents.

PVDF (wt%)	Solvent	Additives ^1^	TiO_2_/PVDF (wt%)	CA (°)	Pore Size (nm)	PWP (Lm^−2^h^−1^bar^−1^)	Ref.
10	NMP	-	0	89	550	770	[43]
		-	10–40	82–88	380–740	1430–480	
12	NMP	-	0	80	N/A	70	[25]
		-	25	64	N/A	150	
12	NMP	-	0	73	200	330	[40]
		-	17	68	120	310	
12	DMAc	PEG	0	78	N/A	300	[27]
			2	74	N/A	290	
15	DMF	-	0	78	43	N/A	[41]
		-	50	81	26–90	N/A	
15	NMP	-	0	87	N/A	303	[42]
		-	30	81	N/A	331	
16	DMF	-	0	78	50.6	88.2	[44]
		-		76	47.3	111.7	
18	DMAc	-	0	N/A	50	77	[13]
		-	8.3	N/A	90	393	
18	DMAc/NMP	PVP	0	79	61	110	[45]
			3–6	65–68	55–115	120–150	
15	NMP	PVP	0	88	N/A	150	[46]
			1.3–27	80–70	N/A	320–180	
18	DMAc	PVP	0	77.1	20.2	67	[47]
			1–3	61–53	64.6–93.9	230–150	
15	PolarClean	F127	0	72	16.8	246	This work
			3.3–13.3	60–52	15.3–12.2	350–288	

^1^ PEG: Polyethylene glycol; PVP: polyvinyl pyrrolidone.

## Data Availability

The original contributions presented in this study are included in the article. Further inquiries can be directed to the corresponding author.
